# American Dental Association

**Published:** 1884-10

**Authors:** Chas. Mayr


### AMERICAN DENTAL ASSOCIATION.

### TWENTY-FOURTH ANNUAL MEETING, AT SARATOGA.

REPORTED FOR THE INDEPENDENT PRACTITIONER BY PROF. CHAS. MAYR.

### TUESDAY EVENING SESSION.

### DISCUSSION OF THE PAPER BY PROF. A. W. HARLAN, UPON PYORRHŒA ALVEOLARIS.

(Concluded from page 524.)

*Dr. Odell*—That is what I call a good paper. The author does not mention manipulation of the gums, and depends upon the drugs to do the whole work. I have had two cases, one quite recent, where manipulation seemed to be the only thing of benefit. This was supplemented by the application of the galvanic current. I came to the conclusion that it was a sort of necrosis.

*Dr. Bogue*—Why does Dr. Harlan recommend Canada Balsam at a certain stage? Can he give the rationale? And why at another point precipitated Chalk and powdered Myrrh?

*Dr. Harlan*—The treatment with Canada Balsam is before the beginning of the systemic treatment; perhaps no particular benefit is derived from the Myrrh in the small quantity used, but it may disguise the taste of other things that might be disagreeable to the patient. The form of a paste seems to have the advantage of not lodging between the teeth.

*Dr. Bogue*—It does not seem consistent after removing deposits to use remedies which, *ex necessitate rei*, must produce deposits. The substances mentioned are precipitated by water, and the effect must be to render the teeth sticky and unclean. I have noticed for many years a tendency to prescribe Tincture of Myrrh, but never found what it was for, and never saw the least benefit from it.

*Dr. Harlan*—I have used the paste myself, and never became conscious of a deposit on my own teeth and gums. I think the patient will brush the paste away much more completely than the powder.

*Dr. Barrett*—With the exception of dental caries, none of the diseases of the teeth come more frequently under our treatment than does Pyorrhœa Alveolaris. Only when we shall understand its etiology will we be enabled to speak intelligently about its therapeutics. Is it an affection of the membranes of the tooth? Is it in the alveolus? Is it confined to the periosteal membrane? Does it arise from constitutional disturbances, or is it merely local and incidental? All these questions must be answered before the practitioner is fully qualified to enter upon its treatment. The cases that have caused me the greatest anxiety have been those in which a simple removal of superficial deposits and the application of astringents, or cauterants, or any other class of remedies, was not sufficient to reach the source of the disease. In one obstinate case, recently under treatment, I was obliged to make repeated surgical operations until I had thoroughly removed nearly all the border of the alveolus about the affected teeth. I believe a concomitant, at least, of severe cases, is a kind of caries of the alveolar edges. It cannot be called a necrosis, for there is no sequestrum; but it is a disintegration of the edges of the septums and walls, due perhaps to a periodontéal inflammation. I never had any success in treatment until I had established a clear line of demarkation; the after treatment was then only palliative. The remedy that I have found most efficacious has been Chloride of Zinc. The stimulating effects of this preparation in the formation of healthy granulations are too well known to need argument. I know of nothing that possesses these virtues in so great a degree. After the surgical operation I usually apply Aromatic Sulphuric Acid in full strength, and the subsequent treatment consists in the application of a solution of Chloride of Zinc in such strength as the case seems to demand.

*Dr. Atkinson*—This subject is highly interesting, because it involves the principles underlying the destruction and reproduction of the tissues. Few of us distinguish sufficiently between an escharotic, a stimulant, and a tonic.

All these agents have the direct influence of coagulating the albuminous portion of the tissues at the particular point, so that, by the operation of the laws of function, new tissues can be formed. But what will occur after this process of coagulation of the albuminous tissues? It is the retrogressive metamorphosis of the layer of tissue immediately underneath that which has been coagulated. The first step is one of retrogressive metamorphosis or return to embryonal conditions, and after this the type produces the tissue belonging to the parts by repeating the action of the functional movements that first resisted destruction.

The point of strength of solutions is very important; twenty grains of Chloride of Zinc to an ounce give that degree of coagulation that will be analogous to that coagulation that takes place in the evolution of the chicken from the impregnated egg, that suited to the fibrillated condition of the lymph. Aromatic Sulphuric Acid and Aqua Regia, one part to seven parts of water, are best suited for that retrogression that is called wasting of the dentinal ligaments, and that is a local disease which occurs many times on only a single tooth; it will start from the margin of the gum, from the point where the dentinal ligament attaches, and go to the end of the root without involving enough to approach the transverse processes, and the teeth most liable are the cuspids. Where there is no deposit, either salivary ceruminal or sanguinary, I doubt the propriety of mechanical interference. I agree, to a certain extent, with Dr. Bogue in what he said about the deposit of Myrrh. When water enough is added to take hold of the alcohol in the solution of Myrrh, it is precipitated.

*Dr. Shepard*—A patient was brought to me by a surgeon; he had a specific disease and was treated for it, and as a result had become troubled with so obstinate a ptyalism that it could not be reduced, and this had lasted for three months. The mercurial treatment had been dropped on account of this salivation. Before the commencement of the mercurial treatment his mouth had been put in order by a prominent dentist. Examining the mouth I found a pronounced case of Pyorrhœa, and the patient was left with me. Pus could be expressed from the margins of the gums in all regions of the mouth, and the discharge was so profuse as to make spots the size of my hand on the pillow at night. By nothing but surgical treatment and a stimulating mouth wash, syringing with Aromatic Sulphuric Acid somewhat diluted under the margin of the gum, he returned to the surgeon in two weeks' time, the gums perfectly well, the pyorrhœa all gone, and the surgeon was enabled to renew the mercurial treatment without a recurrence of the trouble. The deposits under the gum were in this case very slight. I would not discredit the results of Dr. Harlan, but I still think that the thorough removal of the accretions, with cleanliness and syringing with Chloride of Zinc or Aromatic Sulphuric Acid solution, is all that is necessary for treatment.

The essayist was undoubtedly correct, and it should be emphasized still more strongly than by the succeeding speakers, that the salivary calculus and the deposits on the neck which give rise to the affection in question are two different and distinct things; this disease is found upon every tooth in the head where there is never salivary calculus; namely, the palatal surface of the upper molars, the labial surface of the upper incisor, and such positions. I wish we could know something about its etiology. I do not remember to have seen anywhere an explanation of its causes, and our treatment is wholly empirical.

*Dr. Pierce*—The experience which I have had leads me to certain conclusions. I have had many cases of so-called pyorrhœa, but in most of them, when this condition is found in the mouths of children, they are not pyorrhœa, but local inflammation. They present similar features, but readily yield to treatment, and get well. I have never met with a case of urinary calculus without finding two or more teeth affected with pyorrhœa. This fact has led me to believe that the disease is a local expression of a systemic condition, and that the deposit from urine corresponds with accumulations found upon the teeth. We find the disease between the ages of twenty-five or thirty, and forty-five. We find accompanying the disease, if not always preceding it, a filling of the pulp cavity and calcification of the tubuli, and then we find also deposits which resemble tartar on the neck of the teeth. This accumulation precedes the breaking down of the alveolar processes; it precedes the exudation of pus from the alveolar socket, and precedes the inflammatory condition of the gums. Efforts have been made to stop the progress by drilling into the pulp cavity and devitalizing the pulp, but it has not been found an effective treatment. I do not believe that the disease can be cured, but its progress can be modified, and the teeth kept from ten to twenty years by careful treatment. By wiring the teeth to neighboring ones they may be kept in the mouth, but the patient has to come every three months, and the pockets must be thoroughly washed out with Aromatic Sulphuric Acid on a stick shaved down so as to resemble a spatula. This also removes any accumulation on the neck of the teeth. These accumulations I believe to accompany the disease by virtue of the systemic condition, but are not its primary cause. By their presence they act as an exciting cause, stimulating further progress.

*Dr. Rhein*—I have had a number of cases where constitutional treatment of some kind relieved the patient, but after the treatment had stopped a recurrence of the trouble took place. Invariably in these cases a slight deposit was around the neck of the teeth. Very few patients keep the teeth thoroughly clean, and with sufferers from this terrible disease this point becomes imperative.

*Dr. Field*—The assertions of Drs. Harlan and Shepard that there is a marked difference between pyorrhœa and salivary calculus, seem to be something we should fully understand. We know what salivary calculus is; it has been analyzed. But we do not know what the deposit of pyorrhœa is. How can the essayist and my friend from Boston reiterate so confidently that both affections are entirely distinct? I feel more powerless in the treatment of this disease than anything that comes under my hands. I would like to know the distinction; where the one begins and the other ends.

*Dr. Allport*—I consider pyorrhœa entirely constitutional in the large majority of cases, but tartar may be entirely local. In most cases of pyorrhœa alveolaris there are general symptoms of debility. In many cases it is connected with urinary diseases. Bacteria also have an influence, being universally present in the deep pockets. We cannot succeed with local treatment alone. An abscess will recur if it arises from a systemic disturbance, and so will pyorrhœa. Eugenol and Chloride of Zinc are powerful germicides as well as stimulants, and they act beneficially in both ways.

*Dr. James Truman*—Was granted the privilege of the floor, and said: I never heard a more absurd proposition promulgated than that this disease is produced by deposits of tartar. I have endeavored to investigate this matter for the last twenty years—ever since Magitot gave to the disease its name, and when he recommended chromic acid for its treatment. What is pyorrhœa alveolaris? Is it anything more than a local irritation aggravated by systemic conditions? In my judgment it is nothing more than an alveolar abscess translated from the apex to the neck of the tooth ; in other words, an irritation of the pericementum, and as a consequence we have absorption of the alveolus. How is it produced? Not by pressure, if I understand aright. What is pus? It is leucocytes—wandering cells? What are these wandering cells? They are amœba. This was demonstrated years ago by Von Recklinghausen. What is the function of the amœba? It is absorption, and in proportion as we have amœba we have rapid absorption of the tissues.

Adjourned.

